# Tezepelumab for refractory eosinophilic granulomatosis with polyangiitis-related asthma

**DOI:** 10.1186/s12931-024-02888-x

**Published:** 2024-07-11

**Authors:** Nina Vincent-Galtié, Quentin Marquant, Emilie Catherinot, Felix Ackermann, Antoine Magnan, Colas Tcherakian, Matthieu Groh

**Affiliations:** 1 Department of Respiratory Medicine, Hôpital Foch, 40 Rue Worth, Suresnes, 92150 France; 2 Laboratoire VIM-Suresnes, Hôpital Foch, Suresnes, F-92150 France; 3 Université Paris-Saclay, INRAE, UVSQ, VIM, Jouy-en-Josas, 78350 France; 4 Department of Internal Medicine, National Reference Center for Hypereosinophilic Syndrome (CEREO), Hôpital Foch, Suresnes, 92150 France

**Keywords:** Eosinophilic granulomatosis with polyangiitis, Vasculitis, Asthma, TSLP, Tezepelumab

## Abstract

Conventional immunosuppressants are ineffective for the management of EGPA-related asthma. Tezepelumab is a human monoclonal antibody that inhibits thymic stromal lymphopoietin (TLSP) that has proven efficacy in several phase 3 studies for the treatment of asthma. We treated with off-label tezepelumab the first two patients with severe refractory EPGA-related asthma. These preliminary findings suggest that targeting upstream signaling of the T2 inflammatory pathway can improve symptoms, reduce BVAS and increase Asthma Control Test scores, even in patients with refractory asthma who have failed several previous lines of treatment. Nevertheless, by analogy with dupilumab-induced IL-4/13 blockade, the persistence of sputum eosinophilia (reported in both patients) raises questions as to whether TSLP inhibition could lead to a rebound of eosinophilia and potentially to eosinophil-related symptoms in patients with EGPA.

**Supplementary Information:**

The online version contains supplementary material available at 10.1186/s12931-024-02888-x.

Eosinophilic asthma is a hallmark feature of Eosinophilic Granulomatosis with Polyangiitis (EGPA) [1]. Conventional immunosuppressants are ineffective for the management of severe, EGPA-related asthma [2] and persistent airflow obstruction is reported in up to 50% of patients [3]. Tezepelumab is a human monoclonal antibody that inhibits the alarmin thymic stromal lymphopoietin (TLSP) that has proven efficacy in several phase 3 studies for the treatment of asthma [4, 5]. As TSLP is a key upstream driver signal of type 2 inflammation and that variants of *TSLP* (leading to a higher level of TSLP protein secretion) are associated with an increased risk of EGPA [6], we treated with off-label tezepelumab the first two patients with severe refractory EPGA-related asthma despite conventional therapy. This study complied with the MR004 French legislation regarding observational retrospective studies and was approved by Foch Hospital’s independent ethics committee (IRB00012437, approval number 24-01-02).

Case #1: A 73-year-old male patient with a ten-year history of ANCA-negative EGPA complained of persistent severe uncontrolled asthma. Vasculitis remission was obtained thanks to cyclophosphamide induction therapy and azathioprine was then prescribed for maintenance. Seven years later, mepolizumab (100 mg subcutaneously every four weeks) was started due to refractory asthma requiring long-term treatment with oral corticosteroids (OCS). Due to partial efficacy, mepolizumab was then switched to benralizumab (30 mg subcutaneously every four weeks), and OCS were safely tapered. Nevertheless, despite treatment with long-acting β2-agonists, high-dose inhaled corticosteroids and benralizumab, respiratory symptoms worsened four years later (deterioration of lung function, Asthma Control Test ACT 18, three exacerbations requiring short courses of OCS and antibiotics within one year). Benralizumab was stopped and tezepelumab (210 mg every four weeks subcutaneously) was started. After 12 months of follow-up, marked improvement of both ear, nose and throat (ENT) and respiratory symptoms as well as Birmingham Vasculitis Activity Score were reported (Fig. 1). Nevertheless, both a slight increase of absolute blood eosinophil counts (AEC) after 6 months as well as persistent sputum eosinophilia (19%) (Fig. 2) after 12 months were reported, yet without recurrence of respiratory symptoms.

Case #2: The diagnosis of EGPA was retained in 2007 in a 52-year-old female patient with late-onset asthma, hypereosinophilia, polyneuropathy, chronic sinusitis, and pericarditis. ANCA serology was negative. Several lines of treatment including OCS, azathioprine and then mycophenolate mofetil led to the remission of the vasculitis. Mepolizumab and then benralizumab (30 mg subcutaneously every four weeks) enabled weaning of long-term OCS, but asthma control was lost after three years on benralizumab with three asthma exacerbations (including one that required visiting the emergency care department) occurring within a five-month period. Despite partial initial respiratory improvement (Fig. 1) after onset of tezepelumab (210 mg every four weeks subcutaneously), the patient’s condition then progressively worsened to dyspnea at the slightest effort and a hacking cough. Laboratory tests revealed marked sputum eosinophilia (56%) (Fig. 2) while AEC had increased from 0 to 300/mm^3^. A short course of OCS enabled marked clinical improvement, and benralizumab was subsequently resumed.

**Fig. 1:**
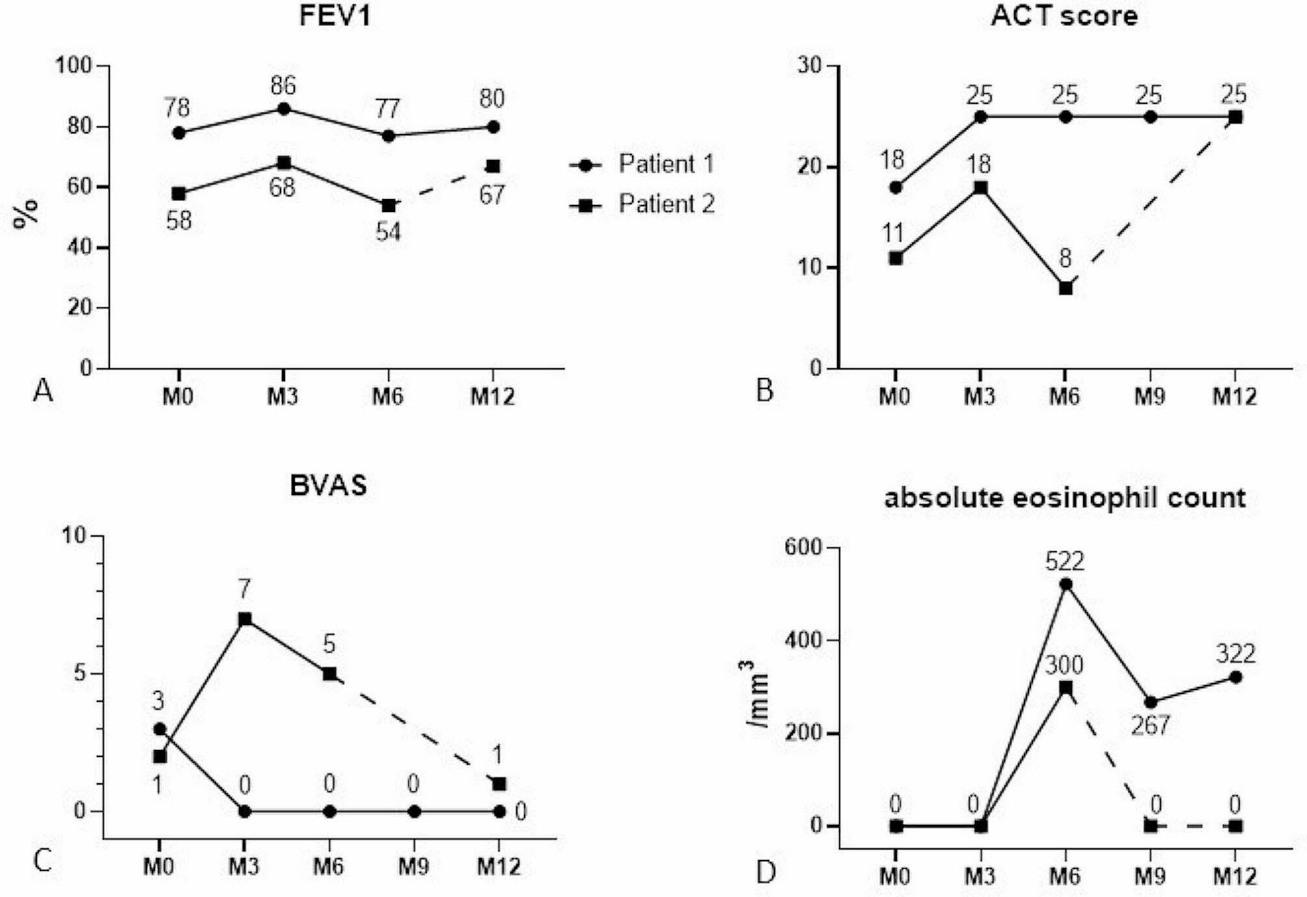
Changes in Forced Expiratory Volume 1 (**A**), Asthma Control Test (**B**), Birmingham Vasculitis Activity scores (**C**) and absolute eosinophil counts (**D**) under treatment with tezepelumab (solid line) or benralizumab (dotted line)

**Fig. 2:**
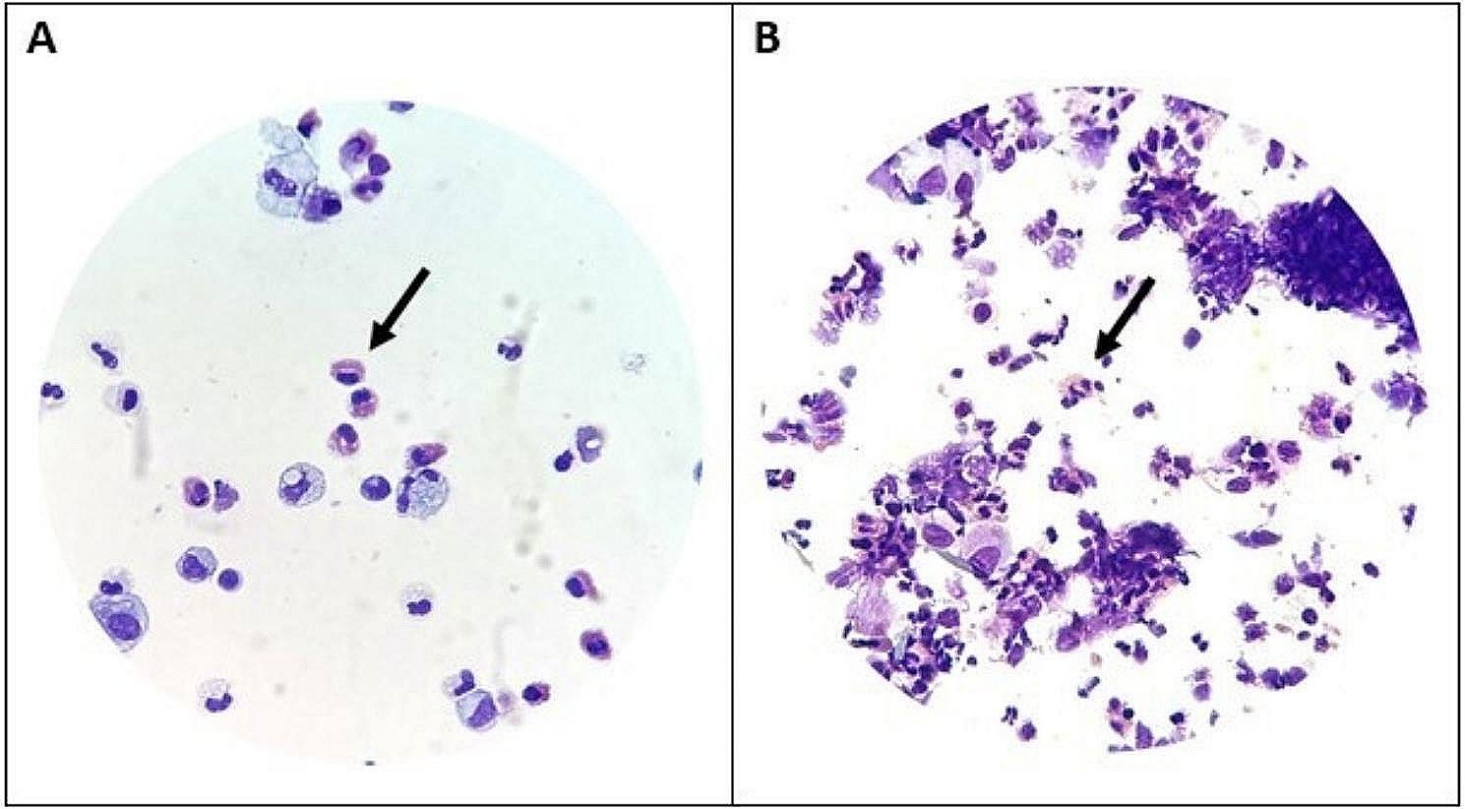
May-Grünwald Giemsa staining from induced sputum of patients #1 (**A**) and #2 (**B**) under treatment with tezepelumab showing mild and marked sputum eosinophilia (arrows), respectively (X80)

Although mepolizumab (a humanized monoclonal antibody that blocks interleukin (IL)-5 from binding to its receptor) is a breakthrough for the management of EGPA, more than half of the patients of the mepolizumab arm in the MIRRA study failed to reach the primary endpoint [7]. Benralizumab – that induces sustained depletion in both blood and tissue of IL-5 receptor alpha-bearing cells – has recently shown to be non-inferior to mepolizumab in EGPA in a head-to-head phase 3 trial [8]. Nevertheless, patients previously treated with mepolizumab are less likely to respond to benralizumab [9]. In a retrospective study of 51 patients, dupilumab (a monoclonal antibody binding to the IL-4 receptor alpha subunit shared by both IL-4 and IL-13 receptor complexes) has proven to be effective for treating both ENT and respiratory symptoms, yet, up to 2/3 of patients developed treatment-induced eosinophilia and 15 (30%) eventually discontinued the drug due to treatment-related adverse events [10]. Taken together these findings stress the need for innovative treatments to curb EGPA-related asthma symptoms.

Here, these preliminary findings suggest that targeting upstream signaling of the T2 inflammatory pathway (with subsequent potential reduction of both T2 cytokines’ levels, T2 innate lymphoid cells and eosinophil activation, Supplemental Figure) can improve symptoms, reduce BVAS and increase Asthma Control Test scores in patients with EGPA with refractory asthma who have failed several previous lines of treatment. Specifically, the marked improvement of case #1’s ENT and respiratory symptoms is promising. Nevertheless, both the evolution of case #2 under treatment as well as the persistence of sputum eosinophilia (despite control of symptoms) in case #1 are intriguing and raise question as to whether TSLP inhibition could, by analogy with dupilumab-induced IL-4/13 blockade, lead to an increase of eosinophils and potentially to eosinophil-related symptoms in EGPA. A potential explanation could be the persistent effects of other T2 alarmins e.g. IL-25 or IL-33 despite TSLP inhibition [11, 12]. Of note, such findings were not reported in phase 3 studies of tezepelumab in asthma outside the scope of EGPA [5, 6]. Likewise, as none of our patients were on OCS on the long run, further studies are warranted to assess the potential OCS-sparing effect of tezepelumab in EGPA. Last, whether the drug is beneficial for ANCA-positive patients and whether it can treat vasculitic symptoms also deserves to be investigated in further prospective studies.

### Electronic supplementary material

Below is the link to the electronic supplementary material.

Supplementary Material 1

## Data Availability

No datasets were generated or analysed during the current study.
