# Supplementary material for: Tezepelumab for refractory eosinophilic granulomatosis with polyangiitis-related asthma
Source: Respir Res. 2024 Jul 11;25:272. doi: 10.1186/s12931-024-02888-x (PMC11242001; doi:10.1186/s12931-024-02888-x)

Supplemental Figure: Tezepelumab improves EGPA symptoms by curbing Type 2 inflammation induced by the secretion of alarmins (*e.g.* TSLP, IL-25, IL-33 and TL1A) by bronchial epithelial cells in response to environmental triggers (created with BioRender.com).


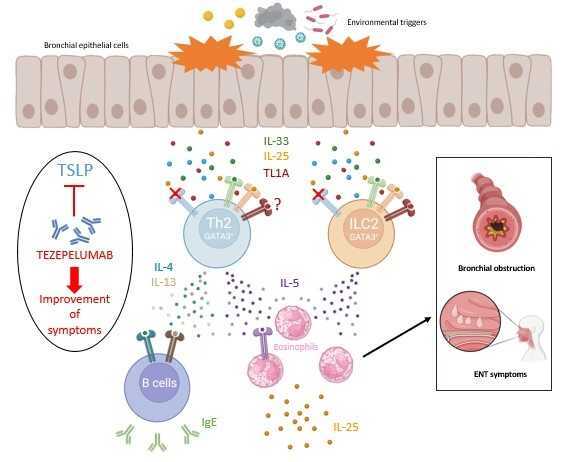

Supplement: Supplementary file 1 — Supplementary Material 1 [file 12931_2024_2888_MOESM1_ESM.docx]
